# Low-flow in aortic valve stenosis patients with reduced ejection fraction does not depend on left ventricular function

**DOI:** 10.1007/s00392-023-02372-4

**Published:** 2024-01-18

**Authors:** Svante Gersch, Torben Lange, Bo Eric Beuthner, Manar Elkenani, Niels Paul, Moritz Schnelle, Elisabeth Zeisberg, Miriam Puls, Gerd Hasenfuß, Andreas Schuster, Karl Toischer

**Affiliations:** 1 Department of Cardiology and Pneumology, University Medical Center Göttingen, Georg-August University, Robert-Koch-Straße 40, 37075 Göttingen, Germany; 2 German Centre for Cardiovascular Research (DZHK), Partner Site Göttingen, Göttingen, Germany; 3 Department of Bioinformatics, University Medical Center Göttingen, Georg-August University, Göttingen, Germany; 4 Department of Clinical Chemistry, University Medical Center Göttingen, Georg-August University, Göttingen, Germany

**Keywords:** Heart failure with reduced ejection fraction, Aortic stenosis, TAVI

## Abstract

**Background:**

Patients with severe aortic stenosis (AS) and reduced left ventricular ejection fraction (LVEF) can be distinguished into high- (HG) and low-gradient (LG) subgroups. However, less is known about their characteristics and underlying (pathophysiological) hemodynamic mechanisms.

**Methods:**

98 AS patients with reduced LVEF were included. Subgroup characteristics were analyzed by a multimodal approach using clinical and histological data, next-generation sequencing (NGS) and applying echocardiography as well as cardiovascular magnetic resonance (CMR) imaging. Biopsy samples were analyzed with respect to fibrosis and mRNA expression profiles.

**Results:**

40 patients were classified as HG-AS and 58 patients as LG-AS. Severity of AS was comparable between the subgroups. Comparison of both subgroups revealed no differences in LVEF (*p* = 0.1), LV mass (*p* = 0.6) or end-diastolic LV diameter (*p* = 0.12). Neither histological (HG: 23.2% vs. LG: 25.6%, *p* = 0.73) and circulating biomarker-based assessment (HG: 2.6 ± 2.2% vs. LG: 3.2 ± 3.1%; *p* = 0.46) of myocardial fibrosis nor global gene expression patterns differed between subgroups. Mitral regurgitation (MR), atrial fibrillation (AF) and impaired right ventricular function (MR: HG: 8% vs. LG: 24%; *p* < 0.001; AF: HG: 30% vs. LG: 51.7%; *p* = 0.03; RVSVi: HG 36.7 vs. LG 31.1 ml/m2, *p* = 0.045; TAPSE: HG 20.2 vs. LG 17.3 mm, *p* = 0.002) were more frequent in LG-AS patients compared to HG-AS. These pathologies could explain the higher mortality of LG vs. HG-AS patients.

**Conclusion:**

In patients with low-flow severe aortic stenosis, low transaortic gradient and cardiac output are not primarily due to LV dysfunction or global changes in gene expression, but may be attributed to other additional cardiac pathologies like mitral regurgitation, atrial fibrillation or right ventricular dysfunction. These factors should also be considered during planning of aortic valve replacement.

**Graphical Abstract:**

Comparison of patients with high-gradient (HG) and low-gradient (LG) aortic stenosis (AS) and reduced ejection fraction. Comprehensive analyses including clinical data, gene expression analyses, cardiovascular magnetic resonance (CMR) imaging as well as echocardiography were performed. AF: Atrial fibrillation, MR: mitral regurgitation, RVEF: right ventricular ejection fraction, ECV%: extracellular volume.

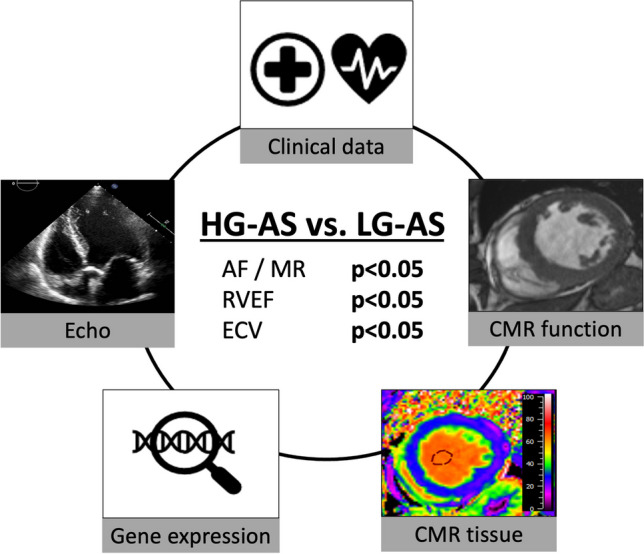

**Supplementary Information:**

The online version contains supplementary material available at 10.1007/s00392-023-02372-4.

## Introduction

Aortic stenosis (AS) is the most prevalent valve disease in the ageing society [1, 2]. Severe AS occurs in 3–5% of individuals over 75 years and is associated with a high burden of morbidity and mortality [3, 4]. AS patients with reduced left ventricular ejection fraction (LVEF) can be subdivided in two groups: 1) AS with reduced LVEF and high transaortic gradient (Vmax > 4.0 m/s or PGmean > 40 mmHg; HG-AS) and 2) AS with reduced LVEF and low gradient (Vmax < 4.0 m/s or PGmean < 40 mmHg; LG-AS).

With evolving evidence, transcatheter aortic valve implantation (TAVI) has become an established treatment method of severe AS during the past decade. While current guideline clearly recommend AVR in symptomatic patients with HG-AS irrespectively from LVEF, diagnosis and treatment of LG-AS is challenging and an extended multimodal diagnostic approach is suggested in order to achieve diagnostic certainty [5]. Importantly, in a previous study, increased myocardial fibrosis (MF) in these subgroups was shown to be associated with poor outcome [6]. Moreover, LG-AS has been demonstrated to be associated with worse prognosis compared to HG-AS even after aortic valve replacement [7, 8]. However, data on HG- and LG-AS phenotypes are scarce and potential underlying hemodynamic mechanisms remain unclear.

Therefore, the aim of this study was to characterize and compare patients with HG-AS and LG-AS in a multimodal approach using clinical data, echocardiography, cardiac magnetic resonance (CMR) imaging, histology and next-generation- sequencing (NGS) for a more in-depth understanding of these heterogenous AS subgroups.

## Methods

Data for this study were gathered at the University Medical Center Göttingen. Patients were classified into a HG and a LG group according to their transvalvular gradient in echocardiography. According to current guideline recommendations HG-AS was defined as a transvalvular gradient > 40 mmHg or a peak aortic jet velocity Vmax > 4 m/s and an aortic valve area (AVA) < 1 cm^2^ and LG-AS with a gradient < 40 mmHg and an AVA < 1 cm^2^, SVI < 35 ml/m^2^. In LG-AS group a pseudo stenosis was ruled out either calcium scoring in computed tomography and/or by stress test. On admission, transthoracic echocardiography was performed, a 6-min walk test, Minnesota Living with Heart failure Quality of life questionnaire (MLHFQ), New York Heart Association (NYHA) status, and N-terminal pro-brain natriuretic peptide (NT-proBNP) levels were measured. Decision for TAVI was based on an interdisciplinary heart team decision according to current guideline recommendations. Additionally, PICP and the CITP:MMP1 ratio, which serves as an indicator of irreversible myocardial fibrosis through collagen cross-linking, was analyzed. A low CITP:MMP1 ratio indicates a presence of irreversible collagen deposition, while a high ratio suggests the possibility of reversible collagen formation [9]. This analysis was conducted using an ELISA-based assay for CITP (Orion Diagnostica, Espoo, Finland) and an alphaLISA method to quantify total serum MMP-1 levels (PerkinElmer, Waltham, Massachusetts, USA) as previously described [10, 11].

The study complied with the Principles of Helsinki and was approved by the local Ethics Committee Göttingen. All patients gave written informed before study participation.

### Echocardiography

**Fig. 1:**
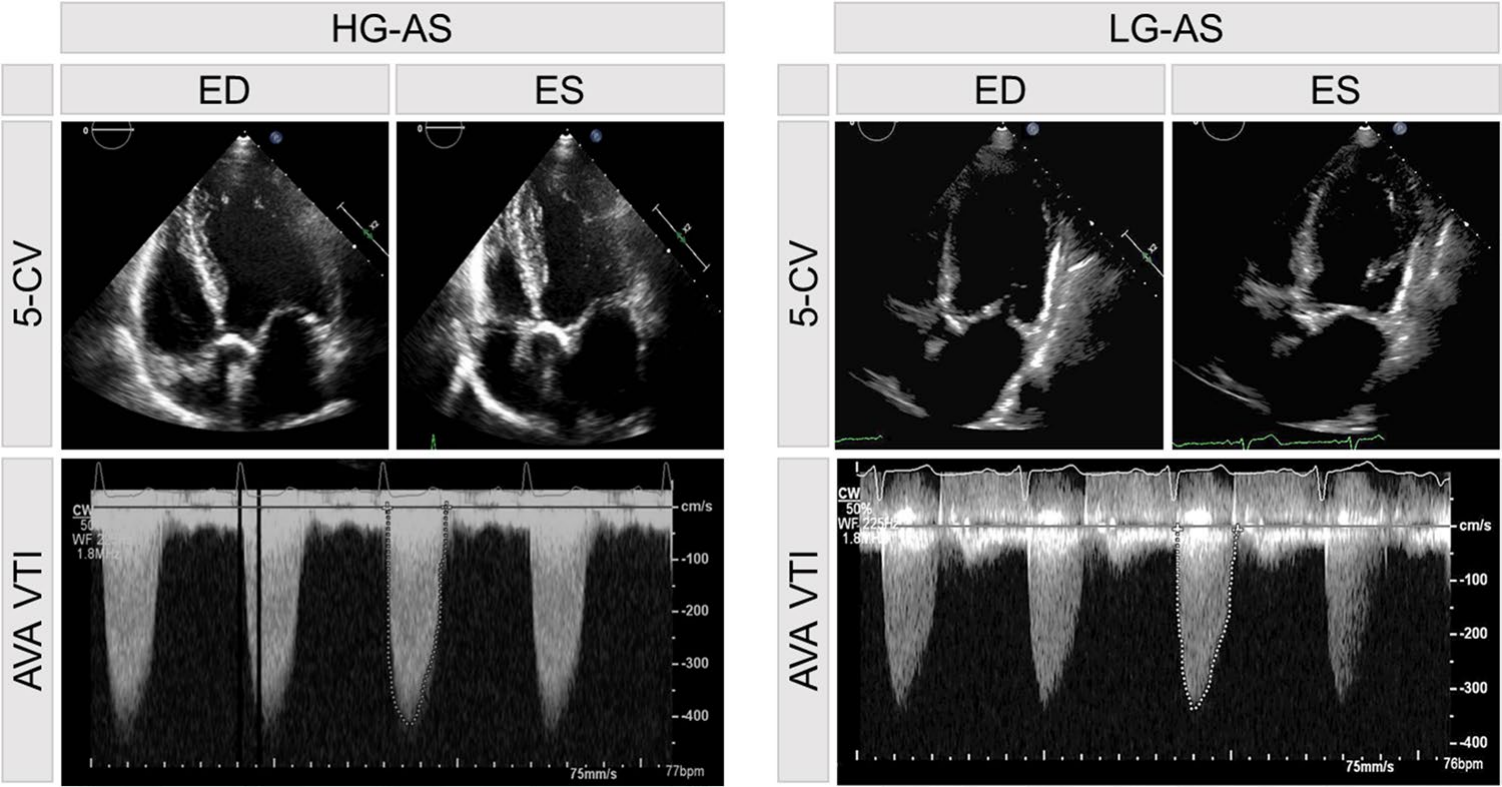
Echocardiographic images of patient with high-gradient (HG) and low-gradient (LG) aortic stenosis (AS), respectively. Pictures in the top showing a 5-chamber view (5-CV) in end-diastole (ED) and end-systole (ES). The corresponding derived aortic valve area velocity time integral (AVA VTI) signal is shown below

As previously described [6], all echocardiographies were performed pre-TAVI and were obtained as recommended [12]. Transvalvular pressure gradient was measured by continuity equation and stroke volume (SV) was computed with pulse wave Doppler signal from apical five-chamber view (Fig. 1). SV was then indexed to body surface area (BSA).

LVEF was calculated by Simpson’s biplane method using the manual tracing of the outline of the endocardial border on apical four and apical two-chamber view. To calculate relative wall thickness the formula (2 × posterior wall thickness/LVEDD) was used. Peak pulmonary artery systolic pressure was calculated using peak tricuspid regurgitation velocity (+ right atrial pressure). In order to validate echocardiographic findings of the study cohort, additional analyses of echocardiographic data obtained from a large clinical all-comer cohort were performed.

### Next generation sequencing

The alignment, normalization and analysis of the RNA-seq data were performed using the RStudio IDE with R version 4.2.2. The raw.fastq files were aligned to the GRCh38 genome using the align function in the Rsubread R package [13]. Only uniquely mapped reads were taken into account. The Rsubread function featureCounts was used to summarize aligned reads to features of the gene annotation file taken from GenCode (Release 40) [14, 15]. After adding a pseudocount of 1 to all features the expression profiles were normalized to GeTMM values [16]. The functions lmFit and eBayes from the limma package were used to check for differentially expressed genes [17, 18].

### Histology

After TAVI procedure, tissue samples were harvested from the basal anteroseptum in the left ventricle using biopsy forceps (Proflex-Bioptom 7F). These samples were then fixed in 10% paraformaldehyde and embedded in paraffin for further analysis. MF was evaluated in a blinded manner, using quantitative morphometry through Olympus Software cell-Sens 1.6. It was defined as the proportion of blue-stained area, indicating collagen presence, in sections of biopsy samples stained with Masson's trichrome. This proportion was determined by comparing the blue-stained area to the total tissue area.

### Cardiovascular magnetic resonance imaging

#### CMR Imaging protocol

**Fig. 2:**
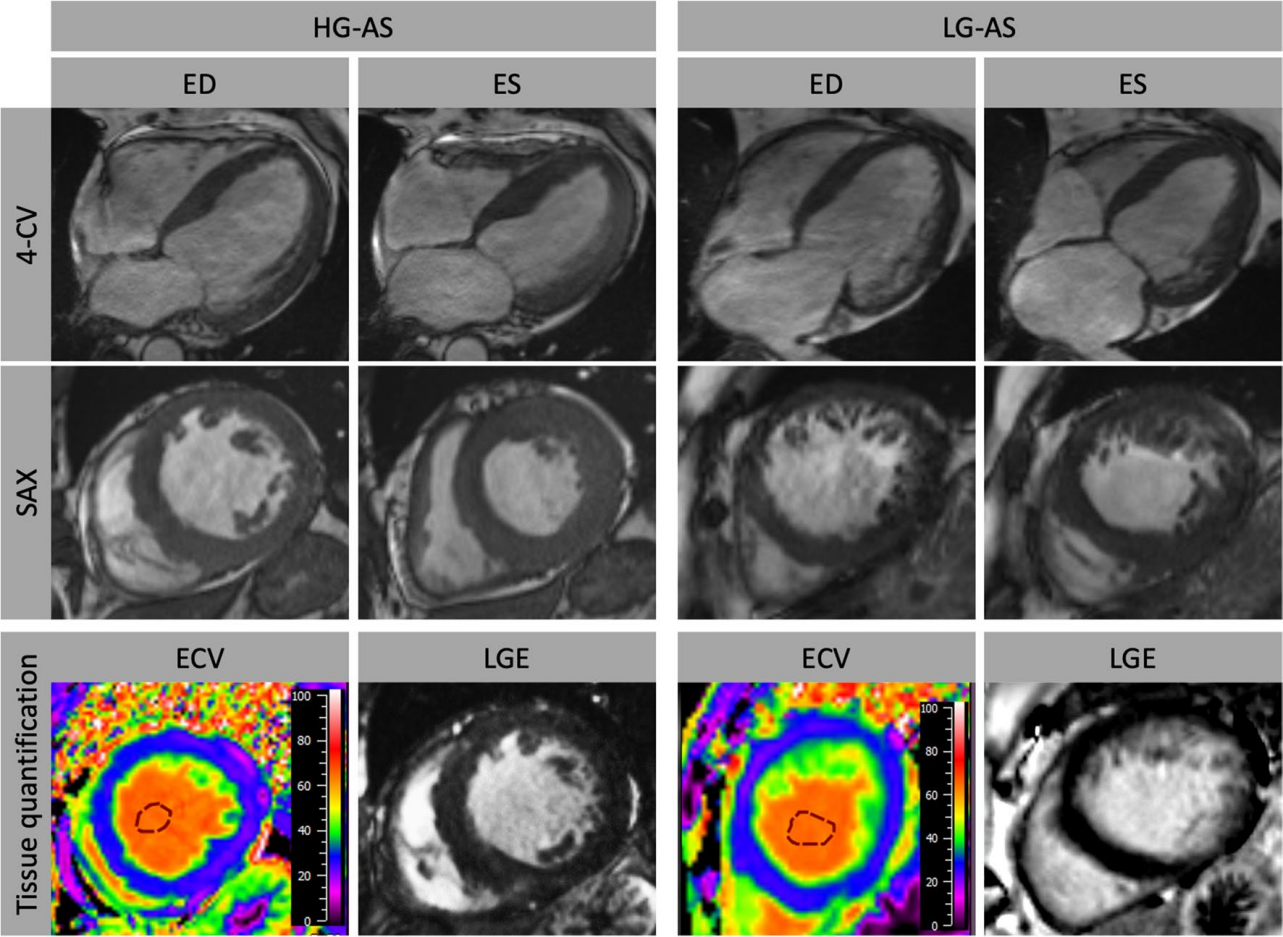
Cardiovascular magnetic resonance imaging in patients with high-gradient (HG) and low-gradient (LG) aortic stenosis (AS). Myocardial function was analyzed in long-axis orientation including 4-chamber view (CV) as well as short-axis (SAX) orientation. Furthermore, tissue characterization comprising calculation of extracellular volume (ECV%) and late gadolinium enhancement (LGE) was performed. ED: end-diastole; ES: end-systole

CMR imaging was performed on a 3.0-Tesla Magnetom Skyra MRI scanner (Siemens Healthcare, Erlangen, Germany) using a 32-channel cardiac surface receiver coil. Electrocardiography-gated balanced steady state free precession (b-SSFP) images of long-axis two, three- and four-chamber views (2-, 3-, 4-CV) as well as short-axis (SAX) stacks were acquired for functional myocardial assessments with the following typical image parameters: 25 frames per cardiac cycle, time of echo (TE) 1.5 ms, time of repetition (TR) 55 ms, flip angle 55°, 7 mm slice thickness with 7.7 mm inter-slice gap. Conventional 5(3)3 Modified Look-Locker Inversion Recovery (MOLLI) sequences (FOV of 360 × 306.6mm^2^, in-plane resolution 1.41 × 1.41 x 8mm^3^, TR 280 ms, TE 1.12 ms, TI 180 ms, flip angle 35°, bandwidth 1085 Hz/pixel with total acquisition of 11 heart beats) for T1-mapping were performed ahead of admission of a gadolinium contrast bolus (0.15 mmol/kg bodyweight) and 20 min after. Phase-sensitive inversion-recovery-gradient echo sequences were acquired for late gadolinium enhancement (LGE) analyses 15–20 min after the gadolinium bolus injection with the following typical imaging parameters: TR 700 ms; TE 1.24 ms; flip angle 40°; slice thickness 7 mm and individually adjusted inversion times typically between 300 and 400 ms) (Fig. 2).

#### CMR image analysis

Based on b-SSFP images strain assessments were performed applying dedicated CMR-feature tracking (CMR-FT) evaluation software (Medis Medical Imaging Systems, Leiden, The Netherlands). Epi- and endocardial borders were manually delineated at end-diastole and -systole in 2-, 3- and 4-CV long axis orientations to analyze global longitudinal strain (GLS). Likewise, atrial endocardial contours were delineated in 2- and 4-CV images for the quantification of LA reservoir (LA Es), conduit (LA Ee) and booster pump strain (LA Ea) as described elsewhere [19]. For ventricular volumetric analyses epi- and endocardial borders were manually delineated in SAX stacks covering the entire LV. T1-mapping based assessment of extracellular volume (ECV%) reflecting myocardial tissue that is not occupied by cells was conducted based on motion-corrected MOLLI sequences. The definition of ECV% was as follows: ECV% = (1 – hematocrit) * [Δ R1 myocardium]/ [ Δ R1 blood] according to guideline recommendations [20]. In all T1 weighted images regions of interest were drawn in excluding any LGE areas and careful visual reevaluation of the delineated regions was performed to avoid partial volume effects due to blood pool or adjacent non-myocardial structures [21]. Using SAX images of inversion recovery sequences, LGE analyses were performed by defining a 3 standard deviations (SD) threshold of signal intensity for the detection of ischemic and non-ischemic LGE enhancement after manual epi- and endocardial border delineation as previously established [22].

#### Statistical analysis

Statistical analysis was performed in Graph Pad prism version 9.0. Continuous variables are presented as mean ± standard deviation. Categorial data were presented as frequency and percentage. Variables were tested for normal distribution by Shapiro-Wilk test. Subgroups were compared by two-sided T-test, one-way analysis of variance or Mann-Whitney-U test, if appropriate. For categorical variables, differences between HG- and LG-group were evaluated by Fisher exact test. A *p*-value of < 0.05 was considered statistically significant.

## Results

### Baseline characteristics

**Fig. 3:**
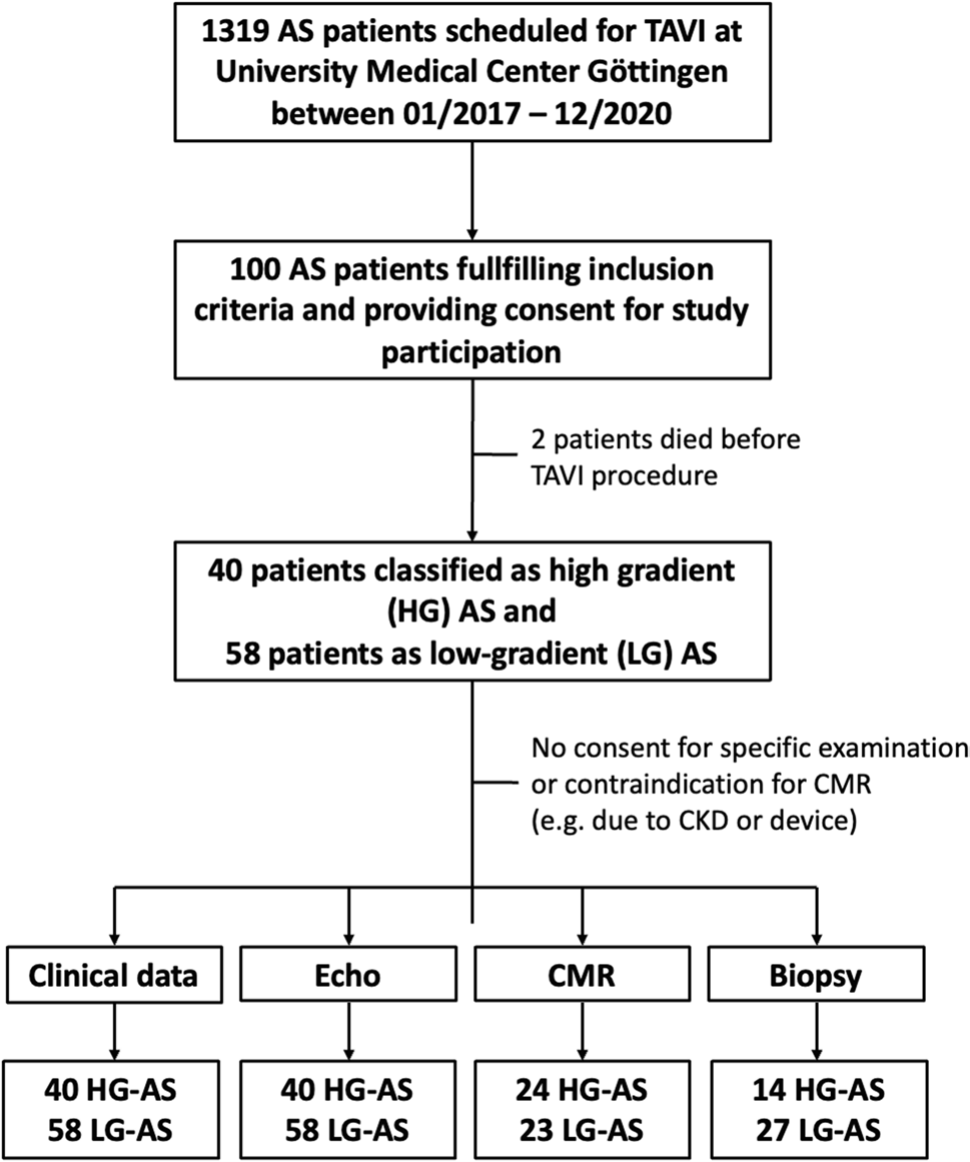
Study flow chart

98 patients with severe AS and impaired left ventricular systolic function, who were scheduled for TAVI at the University Medical Center Göttingen between January 2017 and December 2020 were included to this study (Fig. 3). 40 patients were classified as HG-AS and 58 patients as LG-AS (pseudo stenosis was ruled out either by stress test or calcium score imaging). The mean age of the overall study population was 79.3 (± 6.8) years and participants were predominantly male (75%). Cardiovascular risk factors such as dyslipidemia, arterial hypertension, and diabetes mellitus were present in most of the patients (Table 1). On admission, majority of the patients showed signs of cardiac decompensation coming along with severe symptoms (NYHA III or IV). LG-AS patients showed no significant difference for chronic coronary syndrome (HG: 65% vs. LG: 77.6%; *p* = 0.24), but more prior coronary interventions (HG: 17.5% vs. LG: 44.8%; *p* = 0.005) or coronary artery bypass graft (CABG) (HG: 2.5% vs. LG: 22.4%; *p* = 0.007). Furthermore, atrial fibrillation (AF) occurred more frequently in LG-AS patients (HG: 30% vs. LG: 51.7%; *p* = 0.03), which also correlated with an increased rate of AF on admission (HG: 12.5% vs. 41%; *p* = 0.0029) (Table 2). Neither NT-proBNP (HG: 8707 ± 9109 ng/L vs. LG: 9460 ± 14228 ng/L; *p* = 0.3) nor the 6MWT-distances differed significantly between the two subgroups. (HG: 197.9 ± 130.8 m vs. LG: 189.9 ± 118 m; *p* = 0.77). A more detailed overview of patient characteristics is presented in Table 1.

**Table 1:** Baseline clinical characteristics

Parameters	HG-AS (*n* = 40)	LG-AS (*n* = 58)	*p*-value
Age (years)	79 ± 7.6	79.6 ± 6.1	0.804
Female (n)	13 (32.5)	11 (18.9)	0.154
BMI (kg/m^2^)	26.2 ± 4.9	27.3 ± 5.4	0.446
BSA (m^2^)	1.94 ± 0.2	1.94 ± 0.2	0.896
NYHA I	1 (2.5)	0 (-)	0.408
NYHA II	13 (32.5)	11 (18.9)	0.154
NYHA III	21 (52.5)	37 (63.7)	0.3
NYHA IV	5 (12.5)	10 (17.2)	0.581
Syncope	7 (17.5)	7 (12.0)	0.45
Signs of cardiac decompensation at admission (n)	24 (60)	38 (65.5)	0.671
Cardiac decompensation last 4 weeks (n)	14 (35)	26 (44.8)	0.404
Cardiac decompensation last 6 months (n)	4 (10)	15 (25.8)	0.069
MLHFQ (points)	33.2 ± 15.9	38.5 ± 15.2	0.111
Systolic blood pressure (mmHg)	121 ± 16.4	120 ± 15	0.920
Diastolic blood pressure (mmHg)	69 ± 12.3	66 ± 9.5	0.171
Heart rate (1/min)	72 ± 12.9	73 ± 9.5	0.432
EuroScore 2 (points)	5.0 ± 3.4	10.6 ± 10.3	0.0002
Comobordities			
Diabetes mellitus (n)	13 (32.5)	26 (44.8)	0.294
Arterial hypertension (n)	32 (80)	52 (89.6)	0.242
Dyslipidemia (n)	23 (57.5)	36 (62)	0.679
Smoker (n)	3 (7.5)	10 (17.2)	0.229
Chronic coronary syndrome (n)	26 (65)	45 (77.5)	0.25
Prior myocardial infarction (n)	8 (20)	18 (31)	0.253
Atrial fibrillation / flutter (n)	12 (30)	30 (51.7)	0.039
Prior PCI (n)	7 (17.5)	26 (44.8)	0.005
Prior CABG (n)	1 (2.5)	13 (22.4)	0.007
Aortic valve replacement (n)	0 (-)	1 (1.7)	> 0.999
TAVI (n)	0 (-)	2 (3.4)	> 0.999
Pacemaker / ICD (n)	6 (15)	15 (25.8)	0.222
Peripheral vascular disease (n)	3 (7.5)	15 (25.8)	0.032
eAVK (n)	6 (15)	22 (37.9)	0.022
Prior cerebral ischaemia event (n)	4 (10)	12 (20.6)	0.179
COPD (n)	2 (5)	12 (20.6)	0.039
CHA_2_DS_2_-VASc-Score (points)	5 ± 1.2	5.5 ± 1.3	0.065
6-min walk test, (meters)	197.9 ± 130.8	189.9 ± 118	0.767
Medication			
Angiotensin converting enzyme inhibitor (n)	21 (52.5)	26 (44)	0.539
Angiotensin II receptor antagonist (n)	7 (17.5)	14 (24.1)	0.465
Sacubitril/Valsartan (n)	1 (2.5)	5 (8.6)	0.396
Betablocker (n)	27 (67.5)	41 (70.6)	0.8
Aldosterone antagonist (n)	13 (32.5)	18 (31.0)	> 0.999
Loop diuretic (n)	23 (57.5)	42 (72.4)	0.135
Thiazide (n)	9 (22.5)	11 (18.9)	0.799
Amiodarone (n)	1 (2.5)	3 (5.1)	0.643
Statine (n)	24 (60)	38 (65.5)	0.671
Aspirin (n)	22 (55)	34 (58.6)	0.836
Vitamin K antagonist (n)	6 (15)	13 (22.4)	0.441
NOAC (n)	5 (12.5)	10 (17.2)	0.581
Laboratory			
Haemoglobin (g/dl)	12.5 ± 1.7	12.5 ± 2.01	0.9
NT-proBNP (ng/L)	8707 ± 9109	9460 ± 14,228	0.3
Creatinine (mg/dl)	1.22 ± 0.78	1.49 ± 1.29	0.004
MDRD (ml/min/1.73 m^2^)	62.5 ± 19.2	54.7 ± 22.1	0.1

Baseline characteristics of the study cohort. Continuous variables are expressed as mean ± standard deviation, categorical data are represented as frequency and percentage. Differences between both groups were tested with two-sided t-test or Mann-Whitney-U test

*BMI* body mass index, *BSA* body surface area, *CABG* coronary artery bypass graft, *COPD* chronic obstructive pulmonary disease, *MDRD* modification of diet in renal disease, *ICD* implantable cardioverter defibrillator, *MLHFQ* Minnesota living with heart failure questionnaire, *NOAC* non-vitamin K antagonist anticoagulant, *NT-proBNP* B-type natriuretic peptide, *NYHA* New York Heart Association, *PCI* percutaneous coronary intervention, *TAVI* transcatheter aortic valve implantation

**Table 2:** ECG characteristics

Parameters	HG-AS (*n* = 40)	LG-AS (*n* = 56)	*p*-value
QRS duration	120 ± 35	127 ± 31	0.3
Sinusrhythm at admission	31 (77.5)	27 (48)	0.006
AF/-flutter at admission	5 (12.5)	23 (41)	0.003
RBBB	3 (7.5)	10 (18)	0.22
LBBB	3 (7.5)	9 (16)	0.35
Intraventricular (non-LBBB, non-RBBB)	11 (27.5)	10 (18)	0.32

ECG parameters of the study cohort. Continuous variables are expressed as mean ± standard deviation, categorical data are represented as frequency and percentage

*AF* atrial fibrillation, *LBBB* left bundle branch block, *RBBB* right bundle branch block

### Severity of AS and left ventricular geometry

Echocardiographic characteristics are presented in Table 3. AVA was slightly smaller in HG-patients compared to LG-patients. There was no difference in LVEF (HG: 37 ± 9.7% vs. LG: 33.9 ± 9.2%; *p* = 0.1), LVEDD (HG: 51 ± 8.7 mm vs. LG: 54 ± 7.9 mm, *p* = 0.12), relative wall thickness (HG: 0.54 ± 0.16 vs. LG: 0.48 ± 0.12, *p* = 0.25) or LVMI (HG: 170 ± 36.5 g/m^2^ BSA vs. LG: 166.8 ± 41.4 g/m^2^ BSA; *p* = 0.61). Similar results were found in CMR analyses of these patients (Table 4). These echocardiographic findings were consistent in the additionally analyzed all-comer cohort (Suppl. Table 1).

**Table 3:** Echocardiographic characteristics

	HG-AS (*n* = 39)	LG-AS (*n* = 58)	(*p*-value)
BSA (m^2^)	1.9 ± 0.2	1.9 ± 0.2	0.99
LVEF nS (%)	37 ± 10	33.9 ± 9	0.10
LVEDV (ml)	119 ± 39	122 ± 46	0.71
LVESV (ml)	76 ± 33	83 ± 40	0.39
SVI mD (ml/m^2^ BSA)	34.4 ± 8.4	28.6 ± 6.9	0.0007
LVEDD (mm)	51 ± 8.7	54 ± 7.9	0.12
LVESD (mm)	42 ± 9.0	45 ± 7.9	0.13
IVS (mm)	15 ± 2.1	14 ± 2.7	0.03
PWT (mm)	13 ± 2.4	13 ± 2.4	0.15
LVMI (g/m^2^ BSA)	170 ± 36	166 ± 41	0.61
RWT	0.54 ± 0.16	0.48 ± 0.12	0.25
Vmax (m/s)	4.4 ± 0.4	3.2 ± 0.4	< 0.0001
PGmean (mmHg)	47 ± 9	24 ± 6	< 0.0001
Aortic valve area (cm^2^)	0.61 ± 0.2	0.7 ± 0.2	0.0001
LAVI (ml/m^2^ BSA)	54.7 ± 18	53 ± 17	0.83
TAPSE (mm)	20.2 ± 5.0	17.3 ± 3.7	0.0019
PAP systolic (mmHg)	52 ± 16	47 ± 12	0.13
AR I (n)	19 (49)	25 (43)	0.53
AR II (n)	16 (41)	17	0.28
AR III (n)	0 (0)	0 (0)	> 0.999
MR I (n)	21 (54)	17 (29.3)	0.02
MR II (n)	15 (38)	27 (47)	0.4
MR III (n)	3 (8)	14 (24)	< 0.0001
TR I (n)	22 (56)	30 (52)	0.68
TR II (n)	11 (28)	20 (34)	0.65
TR III (n)	2 (5)	5 (9)	0.69
Stroke volume (ml)	66 ± 14.9	55.6 ± 15.1	0.001
Duration of the flow (ms)	0.35 ± 0.03	0.33 ± 0.03	0.004
Mitral regurgitation volume (ml)	26.7 ± 11.3	32.1 ± 17.6	0.2
SV/ duration of the flow (ml/ms)	186 ± 44	165 ± 42	0.027
SV/ mitral regurgitation volume	2.7 ± 1.1	2.2 ± 1.2	0.12
SV/ mitral regurgitation volume	2.7 ± 1.1	2.2 ± 1.2	0.12
Cardiac output (ml/min)	4.7 ± 1.2	4.0 ± 1.3	0.16

Echocardiographic parameters of the study cohort. Continuous variables are expressed as mean ± standard deviation; categorical data are represented as frequency and percentage

*AR *aortic regurgitation, *BSA* body surface area, *LVEF* left ventricular ejection fraction, *IVS* intraventricular septum, *LAVI* left atrial volume index, *LVEDD* left ventricular end-diastolic diameter, *LVEDV* left ventricular end-diastolic volume, *LVESD* left ventricular end-systolic diameter, *LVESV* left ventricular end-systolic volume, *LVMI* left ventricular mass index, *MI*R mitral regurgitation, *PWT* posterior wall thickness, *RWT* relative wall thickness, *SV* stroke volume, *SVI* stroke volume index, *TAPSE* tricuspid annular plane systolic excursion, *TR* tricuspid regurgitation

**Table 4:** CMR characteristics

Parameter	HG-AS (24)	LG-AS (23)	*p*-value
niLGE volume (ml/ml^2^)	8.9 (5.4–14.9)	7.0 (2.7–13.7)	0.31
iLGE volume (ml/ml^2^)	0.0 (0.0–4.0)	0.0 (0.0–7.2)	0.84
Total LGE volume (ml/ml^2^)	13.2 (7.7–19.3)	7.5 (4.9–21.3)	0.35
ECV% (%)	25.8 (24.4–26.9)	27.1 (26.2–29.6)	0.012
LV matrix volume (ml/ml^2^)	21.5 (18.0–26.3)	22.1 (17.7–25.8)	0.88
LV cellular volume (ml/ml^2^)	65.2 (55.8–75.4)	53.3 (46.0–68.1)	0.16
Total LVMV (ml/ml^2^)	105.0 (90.1–117.8)	90.5 (76.1–109.6)	0.15
LVEDVi (ml/ml^2^)	101.5 (91.5–128.5)	105.3 (80.9–132.9)	0.8
LVESVi (ml/ml^2^)	58.1 (44.9–77.2)	68.2 (40.7–90.1)	0.87
LVSVi (ml/ml^2^)	50.4 (37.5–54.9)	40.7 (34.7–48.7)	0.03
RV volume (ml/ml^2^)	20.8 (16.7–29.8)	22.2 (18.1–26.5)	0.9
RVEDVi (ml/ml^2^)	72.5 (59.7–88.4)	73.3 (58.5–89.4)	0.98
RVESVi (ml/ml^2^)	35.6 (22.4–50.1)	41.3 (31.7–56.5)	0.31
RVSVi (ml/ml^2^)	36.7 (30.1–43.6)	31.1 (24.7–35.8)	0.045
GLS (%)	-15.6 (-12.6- -17.5)	-13.4 (-9.7- -18.3)	0.43
GLS TTP (ms)	373.4 (345.3–426.8)	370.4 (352.5–400.0)	0.83
GCS (%)	-26.7 (-18.0- -34.6)	-20.9 (-14.8- -26.3)	0.19
GRS (%)	40.1 (26.1–51.1)	39.4 (31.4–50.4)	0.87
RV GLS (%)	-23.3 (-15.8- -29.7)	-23.0 (-15.5- -26.2)	0.47
LA Es (%)	11.2 (7.8–16.6)	8.7 (6.9–12.7)	0.21
LA Ee (%)	4.4 (2.7–6.0)	3.8 (1.3–6.6)	0.67
LA Ea (%)	7.4 (4.5–9.4)	5.4 (3.9–9.5)	0.46
RVEF (%)	49.1 (38.1–62.4)	43.7 (32.2–50.8)	0.023
LVEF (%)	43.5 (37.3–53.1)	38.0 (28.4–48.1)	0.17
RA Es (%)	12.8 (10.1–17.4)	12.7 (8.0–14.3)	0.33
RA Ee (%)	6.1 (2.5–9.0)	4.8 (2.0–5.9)	0.14
RA Ea (%)	8.5 (5.8–11.5)	7.0 (4.2–9.3)	0.33
CMR PCWP	16.5 (14.5–21.1)	19.0 (16.0–21.4)	0.32

CMR imaging-derived parameters. Variables are presented as median (interquartile range). Values of high-gradient (HG) and low-gradient (LG) aortic stenosis (AS) patients were compared using Mann-Whitney-U test. *P*-values in bold indicate a statistical significance

*CMR PCWP* pulmonary capillary wedge pressure (CMR-derived), *ECV%* extracellular volume fraction, *GCS* global circumferential strain, *GLS* global longitudinal strain, *GLS TTP* global longitudinal strain time to peak, *GRS* global radial strain, *iLGE* ischemic late-gadolinium-enhancement, *LA Ea* left atrial boosterpump strain, *LA Ee* left atrial conduit strain, *LA Es* left atrial reservoir strain, *LGE* late gadolinium enhancement, *LVEF* left ventricular ejection fraction, *LVEDVi* left ventricular end-diastolic volume index, *LVESVi* left ventricular end-systolic volume index, *LVSVi* left ventricular stroke volume index, *niLGE* non-ischemic late gadolinium enhancement, *RA Ea* right atrial boosterpump strain, *RA Ee* right atrial conduit strain, *RA Es* right atrial reservoir strain, *RVEDVi* right ventricular end-diastolic volume index, *RVEF* right ventricular ejection fraction, *RV GLS* right ventricular global longitudinal strain, *RVESVi* right ventricular end-systolic volume index

The lower stroke volume (HG: 66 ± 14.9 ml vs. LG: 55.6 ± 15.1 ml; *p* = 0.001) in LG-AS patients, however, could be explained by a shorter flow duration via the stenosed aortic valve (HG: 0.35 vs. LG: 0.33; *p* = 0.004). This indicates that despite comparable LVEF and LVEDD the left ventricle cannot generate the same pressure time integral. Characterization of the LV by CMR did not show a difference in left ventricular strain or volumes, while ECV% was significantly higher in LG-AS compared to HG-AS patients (27.1% [26.2–29.6] vs. 25.8 [24.4–26.9], *p* = 0.012) (Table 4). After splitting the two groups according to LVEF below and above 35%, there was an even distribution of valve insufficiencies and left ventricular geometry in the respective subgroups compared to the corresponding group (Table 5).

**Table 5:** Echocardiographic characteristics classified according to LVEF

	HG-AS, EF > 35% (*n* = 28)	HG-AS, EF < 35% (*n* = 11)	LG-AS, EF > 35% (*n* = 29)	LG-AS, EF < 35% (*n* = 29)	*p*-value
BSA (m^2^)	1.9 ± 0.23	2.0 ± 0.24	1.9 ± 0.23	1.9 ± 0.21	0.17
LVEF nS (%)	42 ± 5	24 ± 6 (a)	42 ± 4.0	26 ± 5 (d)	< 0.001
LVEDV (ml)	106 ± 36	153 ± 32 (a)	100 ± 27	144 ± 50 (d)	< 0.001
LVESV (ml)	61 ± 23.3	116 ± 26 (a)	58 ± 18	107 ± 40 (d)	< 0.001
SVI mD (ml/m2 BSA)	36.8 ± 7.1	29.5 ± 5.8 (a)	27.4 ± 6.4	29.7 ± 7.1	< 0.001
LVEDD (mm)	48.5 ± 7	59.8 ± 5 (a)	51.2 ± 7	57.1 ± 7 (d)	< 0.001
LVESD (mm)	38.7 ± 6	52.3 ± 6 (a)	41.7 ± 7	48.6 ± 7 (d)	< 0.001
IVS (mm)	16.1 ± 2.2	15.0 1.9	14.7 ± 3.1	14.5 ± 2.2	0.09
PWT (mm)	13.9 ± 2.0	12.4 ± 2.4	12.6 ± 2.2	12.9 ± 2.5	0.16
LVMI (g/m^2^ BSA)	165 ± 35	189 ± 28	154 ± 33	179 ± 44	0.026
Vmax (m/s)	4.52 ± 0.33	4.2 ± 0.2	3.1 ± 0.38 (b)	3.3 ± 0.46 (c)	< 0.001
Pmean (mmHg)	49 ± 8.3	44 ± 3.4	22.6 ± 5.89 (b)	26.4 ± 6.8 (c)	< 0.001
Aortic valve area (cm2)	0.63 ± 0.17	0.58 ± 0.10	0.7 ± 0.15	0.77 ± 0.16 (c)	0.001
LAVI (ml/m2 BSA)	52.3 ± 16.3	61.4 ± 22.5	51.1 ± 13.5	57.0 ± 19.9	0.34
TAPSE (mm)	21.1 ± 4.9	17.7 ± 4.3	17.5 ± 4.2 (c)	17.0 ± 3.0	0.032
MR III	3 (10)	0	7 (29)	7 (29)	0.47
PAP systolic (mmHg)	49 ± 14.9	62 ± 14.4	47 ± 10.6	48 ± 14.2	0.055
Regurgitation volume (mitral) (ml)	28.1 ± 11.9	22.4 ± 7.9	32.7 ± 15.3	31.7 ± 19.1	0.6
Stroke volume (ml)	67 ± 14	62 ± 15.1	53 ± 15.8	58 ± 13	0.006
Duration of the flow (ms)	0.36 ± 0.03	0.36 ± 0.03	0.33 ± 0.03	0.33 ± 0.03	0.043
SV/ duration of the flow (ml/ms)	190 ± 43	174 ± 45.1	158 ± 43.6	174 ± 39	0.053
SV/ mitral regurgitation volume	2.7 ± 1.1	2.5 ± 0.8	2.0 ± 1.0	2.4 1.1	0.38
Cardiac output	3.9 ± 1.2	4.9 ± 1.7	3.9 ± 1.2	4.2 ± 1.2	0.07

a) HG > 35% vs. HG < 35%; b) HG > 35% vs. LG > 35%; c) HG < 35% vs. LG < 35%; d)LG > 35% vs. LG < 35%

Echocardiographic parameter, classified according to LVEF above and below 35%. Continuous variables are expressed as mean ± standard deviation. categorical data are represented as frequency and percentage

*BSA* body surface area, *LVEF* left-ventricular ejection fraction, *IVS* intraventricular septum, *LAVI* left atrial volume index, *LVEDD* left ventricular end-diastolic diameter, *LVEDV* left ventricular end-diastolic volume, *LVESD* left ventricular end-systolic diameter, *LVESV* left ventricular end-systolic volume, *LVMI* left ventricular mass index, *MR* mitral regurgitation, *PWT* posterior wall thickness, *RWT* relative wall thickness, *SV* stroke volume, *SVI* stroke volume index, *TAPSE* tricuspid annular plane systolic excursion

Notably, a higher prevalence of severe mitral regurgitation (HG: 8% vs. LG: 24%; *p* < 0.001) and an impaired right ventricular dysfunction (RVSVi: 36.7 vs. 31.1 ml/m2; *p* = 0.045; TAPSE: 20.2 vs. 17.3 mm, *p* = 0.002) were detected in the LG-group. Furthermore, impaired hemodynamics especially affecting the right heart might be additionally indicated by regional differences in LV strain. Comparing septal vs. lateral GLS within each group revealed a significantly reduced septal GLS in the LG group (septal GLS -8.2% vs. lateral GLS -12.7%, *p* = 0.008), which might be caused by raised loading pressures in the right ventricle hampering septal contractility (Table 6). In the HG group there was no difference between septal and lateral GLS (septal GLS -11.4% vs. lateral GLS -12.0%, *p* = 0.22).

**Table 6:** Comparison of septal and lateral global longitudinal strain

	Septal	Lateral	*p*-value
HG-AS GLS	−11.4%	−12.0%	0.22
HG-AS GLS TTP	397 ms	385 ms	0.03
LG-AS GLS	−8.2%	−12.7%	0.008
LG-AS GLS TTP	366 ms	369 ms	0.49
	HG-AS	LG-AS	*p*-value
Septal GLS	−11.4%	−8.2%	0.32
Septal GLS TTP	397 ms	366 ms	0.27
Lateral GLS	−12.0%	−12.7%	0.63
Lateral GLS TTP	385 ms	369 ms	0.33

*AS* aortic stenosis, *HG* high-gradient, *GLS* global longitudinal strain, *LG* low-gradient, *TTP* time to peak

**Fig. 4:**
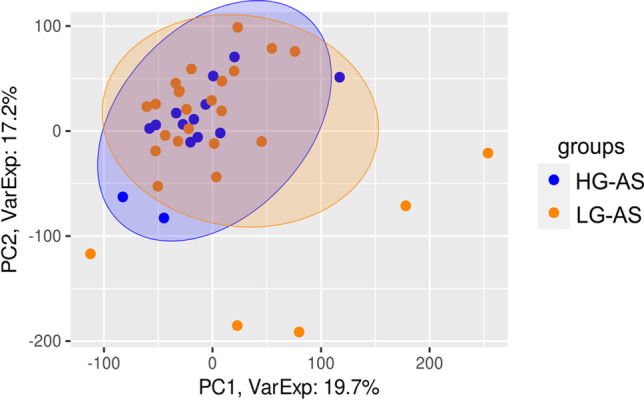
Principal component analysis (PCA) was employed to depict single LV biopsy samples obtained from individual patients. Each circle within the plot represents the projection of data from individual patient samples onto components 1 and 2, along with the percentage of total variance (x/y axis legend). Each patient's dataset is represented by two technical replicates and any outlier replicates have been excluded. (14 HG-AS and 27 LG-AS)

Histology or next-generation sequencing did not show a difference in fibrosis nor global gene expression profile (Fig. 4) between HG- and LG-AS patients. Furthermore, neither PITP-levels (HG: 151 ± 33% vs. LG: 144 ± 40%; *p* = 0.58), nor CITP (HG: 10.8 ± 9.5% vs. LG: 12.3 ± 11.9%; *p* = 0.65) and CIPT/MMP1-ratio differed significantly (HG: 2.6 ± 2.2% vs. LG: 3.2 ± 3.1%; *p* = 0.46). Furthermore, CMR-derived PCWP did not differ between both groups (16.5 [14.5–21.1] vs. 19.0 [16.0–21.4], *p* = 0.32) suggesting no difference in preload induced contractile activation according to the Frank-Starling-mechanism.

## Discussion

This study comprehensively investigated characteristics of severe HG- and LG-AS patients with reduced LVEF and there are some major findings to be considered:

1. There were no significant differences in left ventricular function and volumetric analyses.
2. No differences between subgroups were detected via histological, gene expression and circulating biomarker-based assessment.
3. Right ventricular function was significantly impaired in LG-AS patients compared to HG-AS.
4. Prevalence of atrial fibrillation and high-grade mitral regurgitation was significantly higher in LG-AS patients.

To compensate increased afterload caused by severe AS, the myocardium hypertrophes with subsequent remodeling processes leading to increased myocardial fibrosis. As the disease progresses, compensatory capabilities of the myocardium begin to fail resulting in a decrease of myocardial function [7, 23]. Importantly, patients with severe AS and reduced LVEF have worse outcome than AS patients with preserved LVEF [24]. Nevertheless, there are two different subgroups of AS patients with reduced LVEF – one subgroup generating high transaortic pressure gradients, whereas the other group has only low pressure gradients, suggesting different left ventricular responses on pressure overload. However, there were no significant differences neither in LV function comprising strain and volumetry, nor in histological and NGS assessment between both subgroups. The only LV parameter that significantly differed was CMR-derived ECV%, which is considered as a surrogate parameter for diffuse myocardial fibrosis. Since this is in contrast to the histological and NGS findings, it remains speculative, whether these results are caused by the limitation of a local biopsy, whereas ECV% is more sensitive to detect global remodeling processes [25]. It is noteworthy, that AS is known to exhibit a characteristic diffuse myocardial fibrosis pattern [22, 26]. Therefore, when evaluating myocardial fibrosis using methods like biopsy or CMR, it's crucial to account for factors like location, sampling methods, and technical analysis aspects. Invasive biopsies are limited by size and sampling precision issues, whereas LGE and ECV measurements encompass different myocardial regions, providing complementary information, which should be considered when interpreting the (combination of) different results and parameters.

In contrast, there was a significant reduction in right ventricular function (both in echocardiography and CMR) in the LG-AS subgroup. Following considerations of the Frank-Starling mechanism, RV dysfunction typically develops as a consequence of pressure and/or volume overload. This might be caused by an impaired left heart performance, chronic lung disease or pulmonary hypertension [27]. In addition, the occurrence of LV hypertrophy/LV dysfunction was shown to influence RV function by a pathological downstream response to pressure overload. In this context, in this study documented impaired septal GLS contraction in LG-AS patients might further explain deterioration of RV function.

On the one hand, the current results do not show relevant differences in left heart (dys-)function and consequently one might speculate, whether the deterioration of RVEF is rather caused by an intrinsic RV myopathy than as a mere consequence of LV failure. In line with these considerations, the CMR-derived PCWP did not differ between both subgroups and, therefore, potentially increased LV filling pressures might not explain an impaired RVEF and rather points towards an intrinsic right heart pathology. However, especially the right ventricular and atrial strain values were not reduced in the LG-AS subgroup. Since deformation parameters like CMR-derived strain have been demonstrated to be even more sensitive parameters to detect functional deterioration than volumetric alterations and that functional changes precede myocardial geometric alterations, a fundamental RV contractile deterioration cannot be demonstrated [28, 29]. Therefore, a decreased RVEF might rather be caused by impaired (LV and RV) hemodynamics than by a functional deterioration per se.

On the other hand, further explanations for altered hemodynamics and a lower transaortic gradient might be comorbidities like AF or a higher prevalence of mitral regurgitation, which may result in an incapability of the left ventricle to generate a higher gradient across the stenotic aortic valve. Since the severity of functional MR in AS patients is often directly influenced by elevated systolic ventricular pressures, this might indicate at least slight differences in ventricular pressures/ hemodynamics that are not measurable by the applicated (non-invasive) parameters [30–32].

Importantly, despite pathomechanistical considerations for different gradients, several studies have demonstrated substantial prognostic value both of right ventricular function and comorbidities in AS patients [33, 34]. It is noteworthy that RVD has been demonstrated to be independently associated with adverse outcome after adjusting for other cardiac comorbidities [33]. However, an additional prognostic impact of cardiac comorbidities, such as AF or MR, beyond aortic stenosis and its subsequent impairment of myocardial performance has been shown in several studies [35, 36]. Furthermore, comorbidities like AF have been linked with lower transaortic gradients and with adverse outcome in LG-AS patients, underlining its crucial influence on hemodynamics and prognosis in these patient cohorts [37].

Regarding clinical implications, although guidelines and/or risk calculators include LV function or transaortic gradients for risk stratification and therapeutic decision making in AS patients, they disregard other prognostically important parameters. For example, RV function is not implemented in the EuroSCORE II and STS score despite its reported association with adverse outcome in patients undergoing TAVI [38]. Given the growing prevalence of AS and the subsequent increase in valve replacement procedures, it could be worthwhile to develop a dedicated risk score specifically for AS patients. This score could incorporate and assign weights to the mentioned parameters and comorbidities. Furthermore, such an approach might even include the various AS subtypes and their individual risk profiles.

Hence, by comprehensive analyses of myocardial performance including CMR-derived right heart function as well as coexisting comorbidities, optimized timing of valve replacement and post-procedural monitoring could improve future management of AS patients undergoing TAVI. Further studies are needed to validate these findings and to transfer them into clinical practice.

## Limitations

Data were prospectively collected in our TAVI registry, without excluding comorbidities such as CAD, which makes the study population an all-comer cohort. However, the data were collected monocentrically and retrospectively queried. Furthermore, the global NGS data set showed no distinct difference when comparing the two subgroups, but we cannot exclude the possibility of significant differences within individual genes.

## Conclusion

In severe AS with reduced LVEF, patients with low transaortal gradient do not differ in left ventricular function or tissue composition compared to patients with high transaortal gradients. In contrast, right ventricular function is more restricted in the low gradient subgroup and additional cardiac comorbidities like atrial fibrillation or mitral regurgitation are more frequent than in patients with high transaortic gradients. Possessing additional important prognostic information, future diagnostic algorithms and treatment recommendations might therefore incorporate comprehensive assessment of right heart function and comorbidities for optimized treatment and patient management.

## Supplementary Information

Below is the link to the electronic supplementary material.

Supplemental Table 1 (DOCX 15 KB)

## Data Availability

Data are available from the corresponding author upon reasonable request.

## Abbreviations

AF: Atrial fibrillation
AS: Aortic stenosis
AVR: Aortic valve replacement
CITP: Carboxy-terminal telopeptide of collagen type I
CITP:MMP1: Ratio of serum carboxy-terminal telopeptide of collagen type I to serum matrix metalloproteinase-1
CMR: Cardiovascular magnetic resonance imaging
ECV%: Extracellular volume
HG: High gradient
LG: Low gradient
LGE: Late gadolinium enhancement
LVEDD: Left ventricular end-diastolic diameter
LVEF: Left ventricular ejection fraction
MF: Myocardial fibrosis
MMP1: Matrix metalloproteinase-1
MR: Mitral regurgitation
NGS: Next-generation- sequencing
NT-proBNP: N-terminal pro-brain natriuretic peptide
NYHA: New York Heart Association
PICP: C-terminal propeptide of procollagen type I
Pw: Pulsed wave
RVEF: Right ventricular ejection fraction
TAPSE: Tricuspid annular plane systolic excursion
TAVI: Transcatheter aortic valve implantation

## Declarations

The study was approved by the Ethics Committee of the University Medical Center Göttingen and complied with the Declaration of Helsinki. All individuals gave written informed consent before participating in the study.
