# Supplementary material for: Low-flow in aortic valve stenosis patients with reduced ejection fraction does not depend on left ventricular function
Source: Clin Res Cardiol. 2024 Jan 18;114(11):1455–66. doi: 10.1007/s00392-023-02372-4 (PMC12540571; doi:10.1007/s00392-023-02372-4)
Supplement: Supplementary file 1 — Supplemental Table 1 (DOCX 15 KB) [file 392_2023_2372_MOESM1_ESM.docx]

**Supplemental Table 1:** Echocardiographic parameters assessed by transthoracic echocardiography in a clinical cohort

| **Parameter** | **HG-AS**  **(116)** | **LG-AS**  **(267)** | **p-value** |
| --- | --- | --- | --- |
| EF nS (%) | 34.6 ± 10 | 34 ± 10.3 | 0.61 |
| Vmax (m/s) | 4.5 ± 0.4 | 3.2 ± 0,4 | < 0.001 |
| Pmean (mmHg) | 48.9 ± 12.6 | 24.4 ± 7 | < 0.001 |
| aortic valve area (cm^2^) | 0.58 + 0.16 | 0.73 ± 0.16 | < 0.001 |
| Stroke volume index | 32.9 ± 9 | 27.7 ± 7.2 | < 0.001 |
| LVEDD (mm) | 51.1 ± 8.1 | 51.3 ± 7.9 | 0.83 |
